# CXCR5 identifies stem-like resident memory CD8⁺ T cells enriched for latent EBV specificity in tonsils

**DOI:** 10.1126/sciadv.ady8316

**Published:** 2026-01-07

**Authors:** Olga Rivera Ballesteros, Lisa Rieble, Curtis Cai, Takuya Sekine, Vera Nilsén, Sarah Adamo, Thomas R. Müller, Christian Constantz, Julia Niessl, Eoghann White, YouBeen Ko, Tobias Kammann, Elli Mouchtaridi, Yu Gao, Akhirunnesa Mily, Elisa J. M. Raineri, Christopher Stamper, Anne Marchalot, Nicole Wild, Demi Brownlie, Sian Llewellyn-Lacey, Chris Tibbitt, Jakob Michaëlsson, Nicole Marquardt, Jenny Mjösberg, Carl Jorns, Johan K. Sandberg, Jenny Driving, David A. Price, Marcus Buggert

**Affiliations:** ^1^ Center for Infectious Medicine, Department of Medicine Huddinge, Karolinska Institutet, Stockholm, Sweden.; ^2^ ME Transplantation, Karolinska University Hospital Huddinge, Stockholm, Sweden.; ^3^ Department of Clinical Science, Intervention and Technology, Karolinska Institutet, Stockholm, Sweden.; ^4^ Department of Biomedicine, University and University Hospital Basel, Basel, Switzerland.; ^5^ Institute of Precision Medicine, Hospital of Sun Yat-sen University, Zhongshan 2^nd^ Road, Guangzhou, China.; ^6^ Center for Hematology and Regenerative Medicine, Department of Medicine Huddinge, Karolinska Institutet, Stockholm, Sweden.; ^7^ Division of Infection and Immunity, Cardiff University School of Medicine, University Hospital of Wales, Cardiff, UK.; ^8^ Capio Öron Näsa Hals Globen/Odenplan, Stockholm, Sweden.; ^9^ Systems Immunity Research Institute, Cardiff University School of Medicine, University Hospital of Wales, Cardiff, UK.

## Abstract

CXCR5^+^ CD8^+^ T cells emerged as key mediators of antiviral immunity in the context of chronic infection. However, their functional attributes and tissue distribution remain incompletely defined, especially in relation to antigen specificity. Here, we investigated the anatomical localization and antiviral properties of CXCR5^+^ CD8^+^ T cells across multiple sites throughout the human body, with an emphasis on oropharyngeal lymphoid tissues. Tonsils harbored the highest frequencies of CXCR5^+^ CD8^+^ T cells compared to other tissues, many of which expressed Granzyme K and concurrently displayed tissue residency features, as demonstrated by single cell profiling. Irrespective of clonal identity and virus specificity, CD8^+^ T cells expressed CXCR5 more commonly in tonsils compared to vascular circulation. CXCR5 expression was particularly prominent among tonsil-localized CD8^+^ T cells targeting Epstein-Barr virus (EBV) latent antigens and associated with a PD-1^+^ resident stem-like phenotype. These data identify a CXCR5^+^ tissue-resident memory CD8^+^ T cell subset in human tonsils with a potential role in immune surveillance of EBV.

## Teaser

CXCR5^+^ memory CD8^+^ T cells in tonsils show stem-like resident traits and enrich for latent EBV antigen specificity.

## INTRODUCTION

Memory CD8^+^ T cells are traditionally classified into several subsets based on their common migratory patterns or tissue localization. These include central memory T (T_CM_) cells that recirculate through lymphoid tissues (LTs), effector memory T (T_EM_) cells that patrol nonlymphoid tissues (NLTs) and the bloodstream, and tissue-resident memory T (T_RM_) cells that remain localized in most tissue (*1*). T_RM_ cells, in particular, are essential for rapid immune responses and offer superior protection in mucosal tissues such as the skin, gut, and lungs to pathogen re-encounters (*2*). These cells are also present in LTs, highlighting their ubiquitous presence across almost all human tissues (*3*). T_RM_ cells typically express CD69, often with CD103, and alongside CXCR6, CD49a, and PD-1, and generally lack the lymphoid-homing receptors CCR7 and CD62L (*3*). Such phenotypic diversity suggests that T_RM_ cells are organized into specialized subsets that adapt to the unique microenvironments of different tissues, enabling diverse functional roles tailored to local immune demands.

Secondary lymphoid organs harbor a functionally distinct subset of memory CD8^+^ T cells defined by the expression of CXCR5 (*4*). This chemokine receptor typically positions CD4^+^ T follicular helper (T_FH_) cells within the B cell zone to aid in antibody production (*5*). CXCR5^+^ CD8^+^ T cells, while not fully understood, have been associated with immune control of persistent viral replication in mice infected with lymphocytic choriomeningitis virus (*6*, *7*) and in humans infected with HIV-1 (*8*–*10*). Moreover, chronic antigen stimulation appears to drive the formation of stem-like exhausted CXCR5^+^ CD8^+^ T cells in mice, which respond to PD-1 therapy (*7*). Other studies have suggested that these CD8^+^ T cells potentially mimic the role of T_FH_ cells as facilitators of antibody production (*11*). In a landmark study, CXCR5^+^ CD8^+^ T cells were identified in human tonsils and shown to increase immunoglobulin G production when cultured with B cells in vitro, suggesting a direct helper mechanism (*12*). More recently, it has been suggested that CXCR5^+^ CD8^+^ T cells could support humoral immunity via the production of soluble factors, such as interferon-γ (IFN-γ), interleukin-4 (IL-4), and IL-21 (*13*). However, the contributions of CXCR5^+^ CD8^+^ T cells have also been described in some contexts as negative regulators of antibody production (*14*), possibly reflecting context dependency and functional heterogeneity of this subset.

Many viruses use the oral cavity as an entry point for infection, as exemplified by Epstein-Barr virus (EBV), which is typically transmitted via saliva during childhood. EBV infects most of the global population and establishes persistent viral reservoirs in B cells throughout the body, most notably in tonsil tissue, via a program of latency (*15*). Memory CD8^+^ T cell populations targeting latent and lytic phase antigens are established during primary infection and subsequently operate to control the episodic reactivation of EBV (*16*, *17*). Latent antigen–specific memory CD8^+^ T cell responses are typically directed against epitopes from the EBV nuclear antigen 3 (EBNA3) proteins, whereas lytic antigen–specific memory CD8^+^ T cell responses, which often predominate, are typically directed against the immediate early proteins BRLF1 and BZLF1 and some early proteins and subsequently diversify to target late proteins, such as gp350 (*16*, *18*, *19*). In line with these patterns of immunodominance, latent antigen–specific memory CD8^+^ T cells tend to display an early differentiated phenotype, whereas lytic antigen–specific memory CD8^+^ T cells are more skewed toward a T_EM_ phenotype (*20*, *21*). However, most of these findings were derived from studies of the vascular circulation, which may not be fully representative of the local immune response, given that the latent reservoir of EBV resides primarily in LTs, including tonsils (*19*).

On the basis of these observations, we postulated that CXCR5^+^ CD8^+^ T cells, which localize to LTs (*12*, *22*), could play a key role in immune surveillance against persistent viral infections, including EBV. Accordingly, we used high-dimensional flow cytometry and single-cell sequencing techniques to investigate the clonotypic, functional, phenotypic, and transcriptional properties of CXCR5^+^ memory CD8^+^ T cells in relation to antigen specificity and tissue distribution throughout the human body. Our data show that CXCR5^+^ memory CD8^+^ T cells home to LTs, especially the tonsils, where they display features of stemness and tissue residency. Moreover, these tonsil-localized CXCR5^+^ memory CD8^+^ T cell populations exhibit a discrete functional profile, notably characterized by a lack of cytotoxicity, and preferentially target latent antigens from EBV.

## RESULTS

### CXCR5^+^ CD8^+^ T cells are enriched in tonsils

**Fig. 1.**
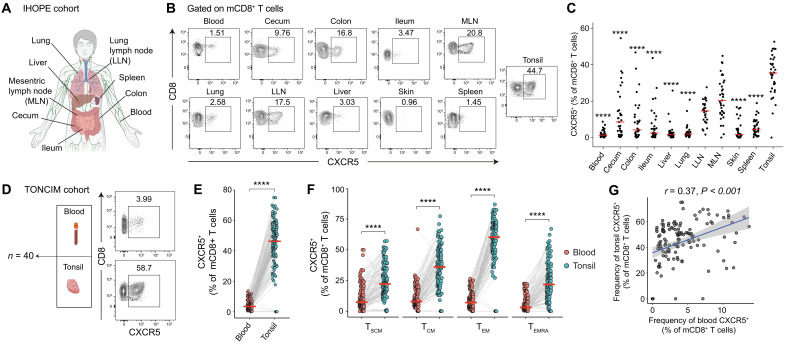
Anatomical distribution of memory CD8^+^ T cells expressing CXCR5. (**A**) Summary of tissues collected as part of the IHOPE cohort. (**B**) Representative flow cytometry plots showing the frequencies of CXCR5^+^ memory CD8^+^ T cells in different tissues. Numbers represent percentages in the drawn gates. (**C**) Dot plot summarizing the frequencies of CXCR5^+^ memory CD8^+^ T cells in different tissues. Each dot represents one donor. Asterisks depict significance relative to tonsil tissue. (**D**) Left: summary of tissues collected as part of the TONCIM cohort. Right: representative flow cytometry plots showing the expression of CXCR5 among memory CD8^+^ T cells in matched blood and tonsil samples. Numbers represent percentages in the drawn gates. (**E**) Paired dot plot showing the frequencies of CXCR5^+^ memory CD8^+^ T cells in matched blood and tonsil samples. Each dot represents one donor. (**F**) Paired dot plot showing the frequencies of CXCR5^+^ T cells among phenotypically distinct populations of memory CD8^+^ T cells in matched blood and tonsil samples. Each dot represents one donor. (**G**) Correlation between the frequencies of CXCR5^+^ memory CD8^+^ T cells in matched blood and tonsil samples with linear regression. The shaded area represents the 95% confidence interval. Significance was assessed using the Mann-Whitney *U* test (C), the Wilcoxon signed-rank test [(E) and (F)], or Spearman’s correlation (G). Horizontal bars indicate median values. ***P < 0.001 and ****P < 0.0001.

To map the anatomical distribution of CXCR5^+^ memory CD8^+^ T cells throughout the human body, we first accessed tonsils, from the TONCIM Tissue Collection Programme, and a broad range of matched tissue samples from a separate cohort of individuals (*n* = 37) recruited as part of the Immunology Human Organ Donor Programme (IHOPE) (*23*, *24*) (Fig. 1A). Although present in all tested tissues, the frequencies of CXCR5^+^ memory CD8^+^ T cells were lower in blood, spleen, and several NLTs, including liver, lung, and skin, compared with lymph nodes and various intestinal sites, including cecum, colon, and ileum, likely reflecting the presence of gut-associated LTs (Fig. 1, B and C). Notably, tonsils harbored the highest frequencies of CXCR5⁺ memory CD8⁺ T cells, consistent with a distinct tissue niche (Fig. 1, B and C).

To extend these findings, we performed similar experiments using matched blood and tonsil samples from a larger cohort of individuals (*n* = 43) recruited as part of the TONCIM Tissue Collection Programme (Fig. 1D). The frequencies of CXCR5^+^ memory CD8^+^ T cells were again substantially higher in tonsils versus blood (Fig. 1E and fig. S1, A and B), and this pattern remained highly significant after stratifying memory CD8^+^ T cells into phenotypically distinct subsets, namely, stem cell–like memory T (T_SCM_) cells (CCR7^+^CD45RA^+^CD95^+^), T_CM_ cells (CCR7^+^CD45RA^−^CD95^+^), T_EM_ cells (CCR7^−^CD45RA^−^CD95^+^), and effector memory RA^+^ T (T_EMRA_) cells (CCR7^−^CD45RA^+^CD95^+^) (Fig. 1F and fig. S1, A and B). CXCR5 expression was especially prevalent among tonsil-localized memory CD8^+^ T cells with a T_EM_ phenotype (Fig. 1F). The frequencies of CXCR5^+^ memory CD8^+^ T cells in tonsils correlated directly with the frequencies of CXCR5^+^ memory CD8^+^ T cells in the vascular circulation (Fig. 1G). Collectively, these results demonstrated that CXCR5^+^ memory CD8^+^ T cells localized primarily to tonsil tissue and, more generally, homed to LTs rather than NLTs.

### Tonsil-localized CXCR5^+^ and CXCR5^−^ memory CD8^+^ T cells are transcriptionally discrete

**Fig. 2.**
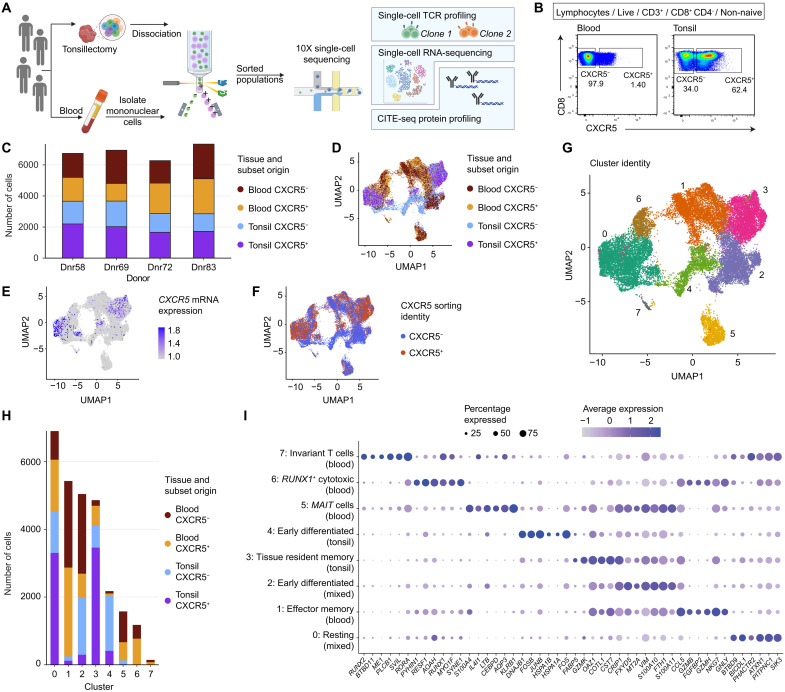
Single-cell phenotypic and transcriptional profiling of CXCR5^+^ and CXCR5^−^ memory CD8^+^ T cells in blood and tonsil tissue. (**A**) Schematic representation of the experimental workflow. (**B**) Gating strategy for the isolation of CXCR5^+^ and CXCR5^−^ memory CD8^+^ T cells via flow cytometry. Numbers represent percentages in the drawn gates. (**C**) Stacked bar chart showing the numbers of cells sequenced per donor by subset and tissue origin. (**D**) UMAP visualization of sequenced cells by subset and tissue origin. (**E**) UMAP visualization of *CXCR5* mRNA expression by subset and tissue origin. (**F**) UMAP visualization of CXCR5 protein expression by subset and tissue origin. (**G**) Cluster analysis via UMAP. (**H**) Stacked bar chart showing of the numbers of cells per cluster by subset and tissue origin. (**I**) Dot plot highlighting differentially expressed genes in each cluster identified via UMAP.

To identify the key features of circulating and tonsil-localized CXCR5^+^ and CXCR5^−^ memory CD8^+^ T cells, we used single-cell sequencing, cellular indexing of transcripts and epitopes sequencing (CITE-seq) in conjunction with T cell receptor sequencing (TCR-seq), to generate comprehensive biological profiles for each cell population (Fig. 2A). Blood and tonsil CXCR5^+^ and CXCR5^−^ memory CD8^+^ T cell populations were sorted from four donors (Fig. 2B), obtaining approximately equal numbers in each case (Fig. 2C). Transcriptional heterogeneity was visualized using dimensionality reduction implemented via uniform manifold approximation and projection (UMAP). Circulating CXCR5^+^ and CXCR5^−^ memory CD8^+^ T cells exhibited many similar transcriptional identities (Fig. 2D), whereas tonsil-localized CXCR5^+^ and CXCR5^−^ memory CD8^+^ T cells segregated into distinct regions, clustering uniquely (Fig. 2D). *CXCR5* expression was enriched among a subset of tonsil-localized CXCR5^+^ memory CD8^+^ T cells, validating our experimental approach (Fig. 2, E and F). When comparing blood and tonsil samples, the median fluorescence intensity of CXCR5 expression was increased in tonsils (fig. S2A). This difference was significant in both the CXCR5⁺ memory CD8⁺ T cell subset and the total memory CD8⁺ T cell population, in tonsil compared with blood, for both control and EBV peptide–stimulated conditions (fig. S2, B and C). Notably, CXCR5 expression showed a considerable overlap with protein expression (by CITE-seq) of CD69 and CD103 (Fig. 2F and fig. S3). Differential gene expression analysis generated eight distinct groups of memory CD8^+^ T cells (Fig. 2G) and revealed that cluster 1 almost exclusively represented T_EM_ cells from the vascular circulation, and that cluster 4, which predominantly incorporated tonsil-localized memory CD8^+^ T cells lacking expression of CXCR5 (Fig. 2H). The top expressed genes and proteins in each cluster (C) identified the major phenotypes as follows: C0, resting (CXCR5^+^) blood and tonsil cells (*ATXN1*, *PITPNC1*, and *SIK3*); C1, effector memory (CXCR5^−^) blood cells (*CCL5*, *NKG7*, and *GZMB*); C2, early differentiated (CXCR5^−^) blood and tonsil cells (*VIM*, *S100A10*, and *FTH1*); C3, tissue-resident memory (protein expression of CXCR5^+^CD69^+^CD103^+^; fig. S2) tonsil cells (*OAZ1*, *CST7*, and *CRIP1*); C4, early differentiated (CXCR5^−^) tonsil cells (*JUNB* and *FOS*); C5, mucosal-associated invariant T (MAIT; CXCR5^−^) cells (*KLRB1* and *S100A4*); C6, RUNX1^+^ cytotoxic (CXCR5^±^) blood cells (*RUNX1*, *CCL5*, and *GZMB*); and C7, invariant (CXCR5^−^) blood T cells (*RORA* and *PHACTR2*) (Fig. 2I and fig. S3). CXCR5^+^ memory CD8^+^ T cells overexpressed genes associated with human stem-like T cell signatures, whereas memory CD8^+^ T cells in blood showed reduced TCF7 and BCL2 expression compared with tonsillar memory CD8^+^ T cells (fig. S4, A and B) (*25*, *26*). Genes up-regulated in CXCR5⁺ memory CD8⁺ T cells were enriched in pathways related to T cell responses and infection, including TCR signaling, antigen processing, and EBV and cytomegalovirus (CMV) infection (fig. S4C), supporting a role for CXCR5⁺ T cells in these processes.

Together, these analyses identified clusters of circulating and tonsil-localized memory CD8^+^ T cells at various stages of differentiation, including early differentiated and cytotoxic effector cells, and revealed transcriptional differences between tonsil-localized memory CD8^+^ T cells segregated by the expression of CXCR5.

### CXCR5 expression among tonsil-localized memory CD8^+^ T cells is associated with a granzyme K–enriched tissue residency profile

**Fig. 3.**
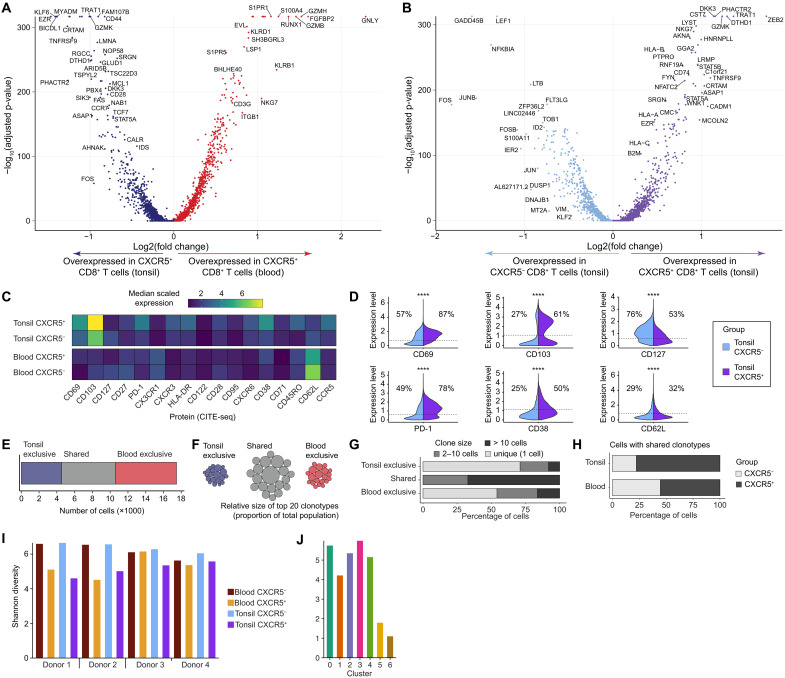
Single-cell clonotypic and transcriptional profiling of memory CD8^+^ T cells expressing CXCR5 in blood and tonsil tissue. (**A**) Volcano plot showing differentially expressed genes between CXCR5^+^ memory CD8^+^ T cells in blood and tonsil tissue. (**B**) Volcano plot showing differentially expressed genes between CXCR5^+^ and CXCR5^−^ memory CD8^+^ T cells in tonsil tissue. (**C**) Heatmap showing median-scaled expression of protein markers by subset and tissue origin measured via CITE-seq. (**D**) Violin plots showing the expression of protein markers by subset among tonsil-localized memory CD8^+^ T cells measured via CITE-seq. (**E**) Stacked bar chart showing the distribution of clonotypes among memory CD8^+^ T cells in blood and/or tonsil tissue. (**F**) Schematic representation of clonal expansions among memory CD8^+^ T cells in blood and/or tonsil tissue. (**G**) Stacked bar chart showing clone sizes among memory CD8^+^ T cells in blood and/or tonsil tissue. (**H**) Stacked bar chart showing the distribution of shared clonotypes among memory CD8^+^ T cells by subset and tissue origin. Significance was assessed using the Mann–Whitney U test with [(A) and (B)] or without Bonferroni correction (D). ****P < 0.0001.

To extend these findings, we analyzed differential gene expression, characterizing memory CD8^+^ T cells segregated by CXCR5 expression and tissue origin. Circulating CXCR5^+^ memory CD8^+^ T cells overexpressed genes encoding effector-like molecules, such as *GZMB*, *GZMH*, *GNLY*, and *NKG7*, as well as egress receptors, such as *S1PR1* and *S1PR5* (Fig. 3A). In contrast, tonsil-localized CXCR5^+^ memory CD8^+^ T cells overexpressed genes encoding memory-like molecules (*GZMK*, *TCF7*, *CD28*, and *CD44*), lymphoid-homing receptors (*EZR* and *CCR7*), and adhesion molecules (*CRTAM*) (Fig. 3A). Moreover, tonsil-localized CXCR5^+^ memory CD8^+^ T cells overexpressed genes associated with a specific effector program (*CST7*, *ZEB2*, and *GZMK*) (Fig. 3B) linked to resident clusters (Fig. 2I), whereas CXCR5^−^ memory CD8^+^ T cells overexpressed genes associated with early differentiation (*LEF1* and *LTB*) and activated phenotypes (*FOS* and *JUNB*) (Fig. 3B), particularly enriched in cluster 4 (Fig. 2I). To confirm some of these transcriptional patterns at the protein level, we next examined specific granzyme expression patterns. CXCR5⁺ memory CD8⁺ T cells expressed granzyme K at significantly higher levels than CXCR5^−^ memory CD8⁺ T cells within the same tissue, whereas granzyme B (GzmB) expression was significantly higher in CXCR5^−^ memory CD8⁺ T cells in blood compared with CXCR5⁺ memory CD8⁺ T cells (fig. S5, A and B). These findings highlight the association of CXCR5 expression with granzyme K in both blood and tissue, and the reduced effector-molecule expression of both CXCR5⁺ and CXCR5^−^ cells in tonsil compared with blood (fig. S5, A and B). Moreover, granzyme K expression correlated with CXCR5 expression within memory CD8⁺ T cells in tonsil, but not in blood, while GzmB expression showed no correlation with CXCR5 expression (fig. S5, C and D).

CITE-seq analyses further revealed that surface expression of protein markers associated with homing (CD62L) and tissue residency (CD69 and CD103) effectively distinguished circulating and tonsil-localized memory CD8^+^ T cells, respectively (Fig. 3C). In addition, tonsil-localized CXCR5^+^ memory CD8^+^ T cells expressed CD69 and CD103 more commonly than tonsil-localized CXCR5^−^ memory CD8^+^ T cells, with a profile indicative of a T_EM_/T_RM_ phenotype (PD-1^+^CD38^+^CD62L^−^CD127^−^) (Fig. 3D). To determine how these phenotypic features evolved over time, we tracked memory CD8^+^ T cells in ex vivo cultures. This revealed significantly lower human leukocyte antigen (HLA)–DR levels and significantly higher expression of residency (CD49a), memory/effector (CD127), and stem-like (TCF1) markers in CXCR5^+^ tonsil-derived memory CD8^+^ T cells by day 7 (fig. S6A). These cells also expressed higher levels of Granzyme K, with a larger fraction coexpressing Granzyme K and B (fig. S6B), consistent with more early differentiated properties.

To assess whether the observed transcriptome and phenotypic features were mirrored in clonal relationships, we analyzed the clonal architecture of memory CD8^+^ T cells segregated by tissue origin, enumerating unique versus shared TCRs (Fig. 3E). The most prominent clonal expansions were shared among circulating and tonsil-localized memory CD8^+^ T cells, both in terms of frequency and size (Fig. 3, F and G). Moreover, these shared clonotypes were enriched among tonsil-localized CXCR5^+^ memory CD8^+^ T cells versus circulating CXCR5^+^ memory CD8^+^ T cells, consistent with a niche preference driven via the TCR (Fig. 3H). Shannon diversity analysis revealed that both CXCR5⁺ and CXCR5^−^ memory CD8⁺ T cells exhibited high levels of TCR repertoire diversity, independent of tissue origin (Fig. 3I). Across individual T cell clusters, repertoire diversity was high for clusters 0 to 4, whereas cluster 5 (MAIT cells from blood) and cluster 6 (RUNX1⁺ cytotoxic T cells from blood) displayed lower diversity (Fig. 3J). Collectively, these results identified CXCR5 as a marker of functionally distinct tissue-resident memory CD8^+^ T cells in tonsil tissue, which shared a degree of clonal identity with memory CD8^+^ T cells in the vascular circulation.

### CXCR5 expression among tonsil-localized memory CD8^+^ T cells is associated with virus specificity

**Fig. 4.**
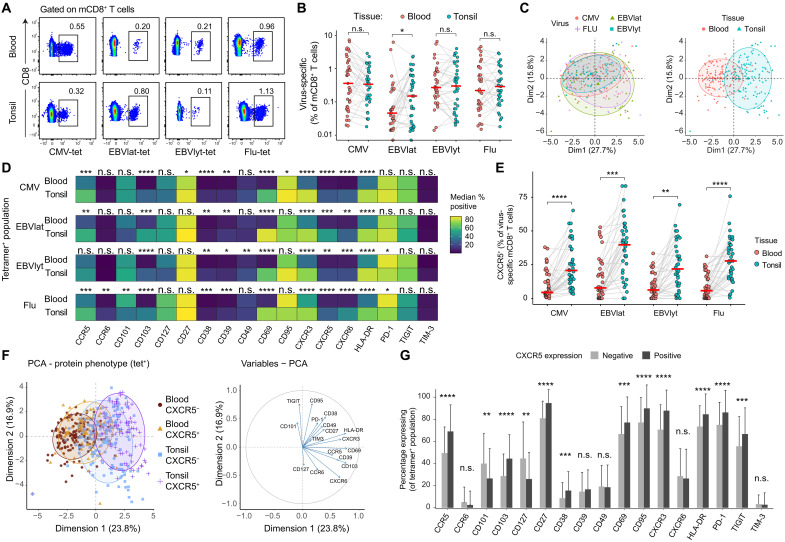
Phenotypic characteristics of virus-specific memory CD8^+^ T cells in blood and tonsil tissue. (**A**) Representative flow cytometry plots showing HLA class I tetramer staining of memory CD8^+^ T cells targeting immunodominant epitopes from CMV, EBV, and influenza virus (Flu) in matched blood and tonsil samples. EBV epitopes were further stratified into latent (lat; EBNA3A and LMP2) or lytic (lyt; BZLF1, BRLF1, and BMLF1) antigens for comparative analyses. Numbers represent percentages in the drawn gates. (**B**) Paired dot plot showing the frequencies of tetramer^+^ memory CD8^+^ T cells in matched blood and tonsil samples. Each dot represents one donor. (**C**) PCA projections showing tetramer^+^ memory CD8^+^ T cells segregated by the expression of phenotypic markers measured via flow cytometry. Populations are colored by specificity (left) or tissue origin (right). (**D**) Heatmap illustrating the median expression of phenotypic markers among tetramer^+^ memory CD8^+^ T cells by specificity, comparing blood versus tonsil tissue. (**E**) Paired dot plot showing the frequencies of tetramer^+^ CXCR5^+^ memory CD8^+^ T cells in matched blood and tonsil samples. Each dot represents one donor. (**F**) PCA projections showing tetramer^+^ memory CD8^+^ T cells segregated by the expression of phenotypic markers measured via flow cytometry. Left: populations are colored by CXCR5 expression and tissue origin. Right: individual markers are shown by relative contribution. (**G**) Bar chart showing the expression of phenotypic markers among tetramer^+^ CXCR5^+^ versus CXCR5^−^ memory CD8^+^ T cells in tonsil tissue. Error bars represent SD. Significance was assessed using the Wilcoxon signed-rank test [(B) and (E)] or the Mann-Whitney *U* test [(D) and (G)]. Horizontal bars indicate median values. *P < 0.05, **P < 0.01, ***P < 0.001, and ****P < 0.0001. n.s., not significant.

Memory CD8⁺ T cells arise following viral infection and persist in tissues, where they play a critical role in controlling viral replication and limiting disease progression upon reinfection. To relate the observed features of CXCR5⁺ T cells in blood and tissues to antigen specificity, we used HLA class I tetramers to identify virus-specific memory CD8^+^ T cells targeting immunodominant epitopes from CMV, EBV, and influenza virus (Flu), representing common, temporally distinct challenges to the immune system (Fig. 4A). Similar frequencies of memory CD8⁺ T cells specific for CMV, EBV lytic antigens, and influenza A virus were detected in blood and tonsils, whereas EBV-specific memory CD8⁺ T cells targeting latent antigens were markedly enriched in tonsil tissue (Fig. 4B). Moreover, the phenotypic characteristics of tetramer^+^ memory CD8^+^ T cells segregated primarily with tissue site rather than virus specificity, as determined via principal components analysis (PCA) (Fig. 4C and fig. S7A). Examining the phenotypic characteristics of tetramer⁺ memory CD8⁺ T cells by virus specificity and anatomical location, we found that tonsil-localized tetramer⁺ memory CD8⁺ T cells in particular expressed CD39, CD69, CD103, CXCR3, CXCR5, CXCR6, and HLA-DR more commonly than circulating tetramer^+^ memory CD8^+^ T cells, irrespective of antigen specificity (Fig. 4D).

Further analyses of CXCR5 expression showed higher frequencies of CXCR5^+^ memory CD8^+^ T cells in tonsils versus blood across each virus specificity (Fig. 4E). To explore these differences in greater depth, we considered the circulating and tonsil-localized tetramer^+^ CXCR5^+^ and CXCR5^−^ memory CD8^+^ T cell populations separately, concatenating phenotypic markers for each of the four subsets and projecting the data via PCA. The most pronounced contrasts were observed between circulating tetramer^+^ CXCR5^−^ memory CD8^+^ T cells and tonsil-localized tetramer^+^ CXCR5^+^ memory CD8^+^ T cells, driven across the first dimension by the expression of CCR5, CD39, CD69, CD103, CXCR3, and HLA-DR (Fig. 4F and fig. S7, B and C). Paired analyses of individual markers further showed that tonsil-localized tetramer^+^ CXCR5^+^ memory CD8^+^ T cells more frequently expressed CCR5, CD27, CD38, CD69, CD95, CD103, CXCR3, HLA-DR, PD-1, and TIGIT, whereas tonsil-localized tetramer^+^ CXCR5^−^ memory CD8^+^ T cells more frequently expressed CD101 and CD127 (Fig. 4G). Together, these data show that virus-specific memory CD8⁺ T cells were present in both blood and tonsils, with EBV latent antigen–specific cells enriched in tonsils, where CXCR5⁺ subsets exhibited a distinct residency-associated phenotype.

### EBV-specific CXCR5^+^ memory CD8^+^ T cells in tonsils exhibit a noncytotoxic functional profile

**Fig. 5.**
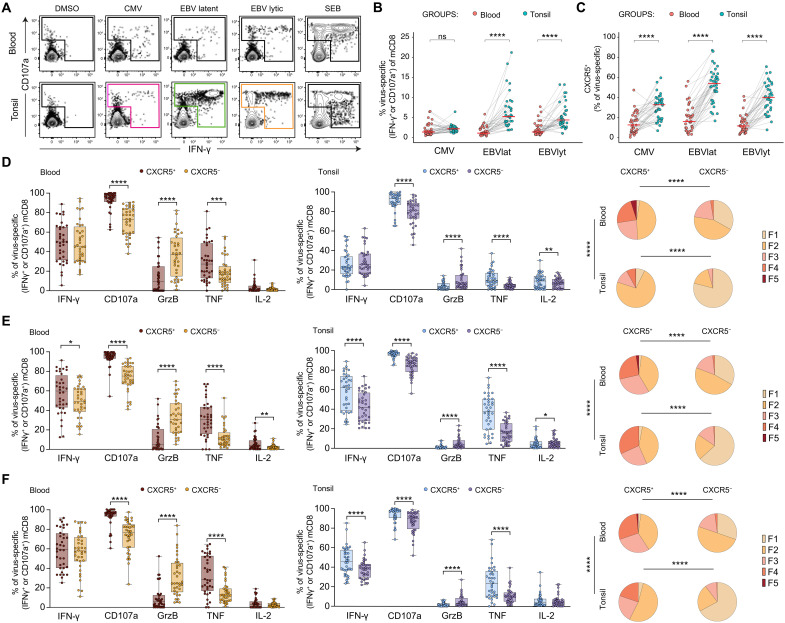
Functional profiles of virus-specific memory CD8^+^ T cells in blood and tonsil tissue. (**A**) Representative flow cytometry plots showing the identification of functional virus-specific memory CD8⁺ T cells in matched blood and tonsil samples following stimulation with CMV peptide pools (IE-1 and pp65) or EBV peptide pools classified as latent (EBNA3A and LMP2) or lytic (BZLF1, BRLF1, and gp350). (**B**) Paired dot plot showing the frequencies of functional (CD107a^+^ or IFN-γ^+^) virus-specific memory CD8^+^ T cells in matched blood and tonsil samples. Each dot represents one donor. (**C**) Paired dot plot showing the frequencies of functional (CD107a^+^ or IFN-γ^+^) virus-specific CXCR5^+^ memory CD8^+^ T cells in matched blood and tonsil samples. Each dot represents one donor. (**D** to **F**) Plots showing the frequencies of functional (CD107a^+^ or IFN-γ^+^) virus-specific CXCR5^+^ or CXCR5^−^ memory CD8^+^ T cells expressing IFN-γ, CD107a, GrzB, TNF, or IL-2 in blood and tonsil samples. Each dot represents one donor, horizontal bars indicate mean values, and significance was assessed using the Wilcoxon signed-rank test. Pie graphs represent the proportion of reactive CXCR5^+^ or CXCR5^−^ memory CD8^+^ expressing one (F1), two (F2), three (F3), four (F4), or all five (F5) cytokines. Significance was assessed with a permutation test. Cells were stimulated with CMV (D), EBV latent (E), or EBV lytic (F) peptides. *P < 0.05, **P < 0.01, ***P < 0.001, and ****P < 0.0001. DMSO, dimethyl sulfoxide; n.s., not significant; SEB, staphylococcal enterotoxin B.

To determine whether virus-specific memory CD8⁺ T cells exhibit distinct functional capacities depending on tissue location and CXCR5 expression, we stimulated paired samples with overlapping peptide pools representing immunodominant proteins from CMV and EBV, with the latter divided into latent and lytic antigens. Antigen-specific memory CD8^+^ T cell responses directed against each peptide pool were readily identifiable in blood and tonsil tissue via concurrent surface mobilization of CD107a or intracellular expression of IFN-γ (Fig. 5A). We detected higher frequencies of EBV-specific memory CD8^+^ T cells in tonsils versus blood following stimulation with both EBV latent and lytic peptides (Fig. 5B), and higher frequencies of responsive memory CD8^+^ T cells expressed CXCR5 in tonsils versus blood across CMV and EBV virus specificities (Fig. 5C).

To further investigate virus-specific memory CD8^+^ T cell response upon peptide stimulation, we quantified the intracellular expression of tumor necrosis factor (TNF), GzmB, and IL-2 alongside surface expression of CXCR5 among responsive (CD107a⁺ or IFN-γ⁺) memory CD8⁺ T cells for each virus specificity (Fig. 5, D and F). Responsive CXCR5⁺ memory CD8⁺ T cells more frequently expressed CD107a and TNF, and less frequently expressed GzmB, compared to their CXCR5^−^ counterparts, regardless of anatomical location or virus specificity (Fig. 5, D and F). IFN-γ expression was higher in CXCR5⁺ memory CD8⁺ T cells specific for EBV latent antigens in both blood and tonsils, and in EBV lytic antigen–responsive CXCR5⁺ memory CD8⁺ T cells in tonsils, indicating that lytic reactivation is primarily occurring in lymphoid tissues (Fig. 5, D to F). Both circulating and tonsil-localized responsive CXCR5⁺ and CXCR5^−^ memory CD8⁺ T cells rarely expressed IL-2 (Fig. 5, D to F). When analyzing functional cytokine profiles, a significantly higher proportion of CXCR5⁺ memory CD8⁺ T cells expressed multiple cytokines compared to CXCR5^−^ memory CD8⁺ T cells, independent of tissue type or antigen specificity (Fig. 5, D to F). Circulating cells also exhibited a greater frequency of polyfunctional cells than tonsil-localized cells. Along with higher GzmB expression observed in circulating CXCR5⁺ and CXCR5^−^ memory CD8⁺ T cells compared to their tonsillar counterparts, these findings support the notion that cytotoxic functionality is predominantly associated with the vascular compartment (*1*, *27*) (Fig. 5, D to F, and fig. S8). Collectively, these findings demonstrated that functional, EBV-specific memory CD8^+^ T cell responses were enriched in tonsil tissue, where a largely noncytotoxic profile segregated with the expression of CXCR5.

### EBV latent antigen–specific memory CD8^+^ T cells in tonsils exhibit a tissue-resident phenotype enriched for expression of CXCR5

**Fig. 6.**
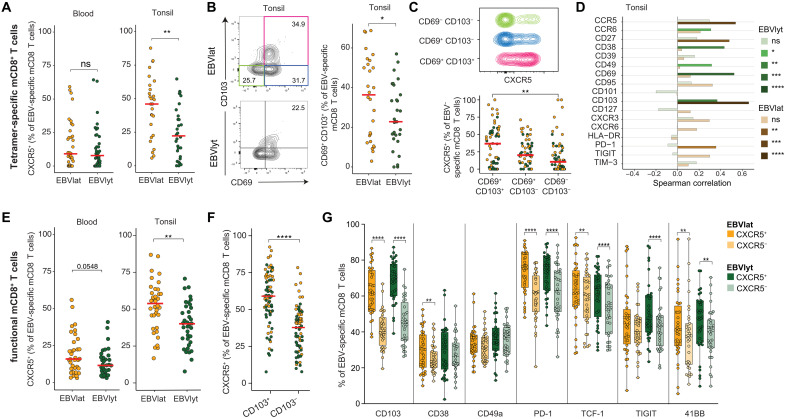
Phenotypic characteristics of EBV-specific memory CD8^+^ T cells targeting latent or lytic antigens in blood and tonsil tissue. (**A**) Dot plots show EBV antigen-specific (tetramer⁺) CXCR5⁺ memory CD8⁺ T cell frequency in blood and tonsil tissue, stratified as latent (EBNA3A and LMP2)– or lytic (BZLF1, BRLF1, and BMLF1)–derived epitopes. Each dot represents one donor. (**B**) Left: representative flow cytometry plots of identification of CD69^+^CD103^+^ EBV latent or lytic antigen–specific (tetramer^+^) memory CD8^+^ T cells in tonsil tissue. Numbers represent percentages within drawn gates. Right: frequencies of CD69^+^CD103^+^ EBV latent or lytic antigen–specific (tetramer^+^) memory CD8^+^ T cells in tonsil tissue. (**C**) Top: representative flow cytometry plot showing CXCR5 expression among EBV latent antigen–specific (tetramer^+^) memory CD8^+^ T cells in tonsil tissue, defined phenotypically as in (B). Bottom: frequencies of EBV latent or lytic antigen–specific (tetramer^+^) CXCR5^+^ memory CD8^+^ T cells among phenotypically distinct subsets in tonsil tissue, as defined in (B). (**D**) Correlations between the expression frequencies of CXCR5 and other phenotypic markers among EBV latent or lytic antigen–specific (tetramer^+^) memory CD8^+^ T cells in tonsil tissue. Colors indicate significance (key). (**E** and **F**) Frequencies of functional (CD107a^+^ or IFN-γ^+^) EBV latent or lytic antigen–specific CXCR5^+^ memory CD8^+^ T cells (E) in blood and tonsil tissue and (F) among phenotypically distinct subsets in tonsil tissue defined by the expression of CD103. Each dot represents one donor. (**G**) Expression frequencies of individual phenotypic markers among functional (CD107a^+^ or IFN-γ^+^) EBV latent or lytic antigen–specific CXCR5^+^ or CXCR5^−^ memory CD8^+^ T cells in tonsil tissue, horizontal bar indicates mean values. Significance was assessed using the Mann-Whitney *U* test [(B), (C), (E), and (F)], Wilcoxon signed-rank test (G), or Spearman’s correlation (D). Horizontal bars indicate median values. *P < 0.05, **P < 0.01, ***P < 0.001, and ****P < 0.0001.

To refine the phenotypic features of virus-specific memory CD8⁺ T cells, we directly compared tetramer⁺ populations targeting latent versus lytic EBV antigens. Comparable frequencies of circulating latent versus lytic antigen–specific memory CD8^+^ T cells expressed CXCR5, whereas higher frequencies of tonsil-localized latent versus lytic antigen–specific memory CD8^+^ T cells expressed CXCR5 (Fig. 6A). Tonsil-localized latent antigen–specific memory CD8^+^ T cells were also enriched for surface expression of residency markers CD69 and CD103 (Fig. 6B). In line with these observations, tonsil-localized memory CD8^+^ T cells with a tissue-resident phenotype (CD69^+^CD103^+^) expressed CXCR5 more commonly than tonsil-localized memory CD8^+^ T cells with a non–tissue-resident phenotype (CD69^−^CD103^−^), irrespective of latent versus lytic antigen specificity (Fig. 6C). Moreover, tonsil-localized latent antigen–specific CXCR5^+^ memory CD8^+^ T cells typically expressed CD27, a marker of early differentiation, alongside CD103 (Fig. 6D).

Stratifying the data from our functional assays for CXCR5 expression, we showed that higher frequencies of responsive (CD107a^+^ or IFN-γ^+^) latent compared to lytic antigen–specific memory CD8^+^ T cells expressed CXCR5, irrespective of anatomical location (Fig. 6E). CXCR5 was also expressed at higher frequencies among tonsil-localized EBV-reactive CD103^+^ versus CD103^−^ memory CD8^+^ T cells after EBV peptide stimulation (Fig. 6F). In addition, higher frequencies of tonsil-localized EBV-reactive CXCR5^+^ versus CXCR5^−^ memory CD8^+^ T cells expressed CD103 and PD-1, as well as the stemness factor TCF-1 and activation marker 41BB (Fig. 6G). Collectively, these results showed that tonsil-localized EBV-specific memory CD8^+^ T cells, especially those specific for latent antigens, typically exhibited a follicular tissue-resident phenotype, potentially contributing to immune control of EBV.

## DISCUSSION

In this study, we used high-dimensional flow cytometry and single-cell sequencing techniques, namely, CITE-seq and TCR-seq, to characterize CXCR5^+^ memory CD8^+^ T cells throughout peripheral and oropharyngeal lymphoid tissues. Our work was enabled by access to curated samples from deceased organ donors and patients undergoing tonsillectomy. We found that CXCR5^+^ memory CD8^+^ T cells were enriched in LTs, especially the tonsils. Tonsil-localized CXCR5^+^ memory CD8^+^ T cells were functionally discrete, lacking classical markers of cytotoxicity, and preferentially targeted latent antigens from EBV. These cells also displayed hallmarks of stem-like tissue residency. Accordingly, tonsil-localized CXCR5^+^ memory CD8^+^ T cells are fully equipped and optimally positioned to combat ingested or inhaled pathogens at the site of entry and protect against persistent viruses with an immune cell tropism, such as EBV.

One of our initial observations was that CXCR5^+^ memory CD8^+^ T cells localized not only to secondary lymphoid organs, such as LNs, but also to LTs, including gut-associated LTs, which has not been reported previously. This finding was made possible by the use of optimized protocols for tissue preparation that preserved the expression of CXCR5 (*28*). We also found that CXCR5^+^ memory CD8^+^ T cells were highly enriched in tonsil tissue, consistent with earlier work (*12*). A similar distribution has been described for germinal center T_FH_ cells, which also express CXCR5 (*29*). This strategic location likely reflects increased antigen presentation in tonsil tissue, which contains higher densities of lymphoid follicles and germinal centers than lymph nodes (*29*–*31*), creating an environment conducive for cellular interactions to facilitate the generation and maintenance of humoral immunity (*32*). In addition, we found that tonsil-localized CXCR5^+^ memory CD8^+^ T cells expressed CD69 and CD103 (*1*, *3*) and down-regulated egress receptors that help limit recirculation, including S1PR1 (*27*), consistent with a tissue-resident phenotype (*33*, *34*). It is notable here that tonsil-localized CD8^+^ T_RM_ cells, unlike lymph node–localized CD8^+^ T_RM_ cells, express CD103 (*35*–*38*). This particular feature could reflect the proximity of epithelial barriers in the oropharynx, further highlighting the sentinel-like nature of CXCR5^+^ memory CD8^+^ T cells in tonsil tissue (*33*, *39*).

Another notable observation was that CXCR5 expression was broadly imprinted among memory CD8^+^ T cells in tonsil tissue, irrespective of antigen specificity or clonal identity. Tonsils fulfill a unique role as the primary lymphoid site for many pathogens as they enter the body. It therefore seems likely that frequent antigen exposure drives the increased prevalence of lymphoid follicles and germinal centers in tonsils versus other secondary lymphoid organs, promoting the expression of CXCR5 (*37*). Expression of this chemokine receptor prolongs interactions with antigen-presenting cells and stromal cells that are crucial for memory retention (*40*). Tonsil-localized CXCR5^+^ memory CD8^+^ T cells also exhibited a less cytotoxic functional profile and a more granzyme K^+^ stem-like phenotypic and transcriptional profile than circulating CXCR5^+^ memory CD8^+^ T cells or tonsil-localized CXCR5^−^ memory CD8^+^ T cells, underscored by the expression of *BCL2* and *TCF7*. These features may represent adaptations to the tonsillar microenvironment that could enable broadly directed immune responses without causing excessive tissue damage. In broader terms, tonsillar CXCR5⁺ CD8⁺ T cells resemble the “stem-like” PD-1⁺TCF1⁺CXCR5⁺ CD8⁺ populations described in chronic viral infections and cancer. These subsets have been shown to sustain exhausted CD8⁺ T cell responses and act as resource cells for generating effectors upon checkpoint blockade. The identification of such a population in human oropharyngeal lymphoid tissue highlights the convergence of stem-like and resident memory programs, and suggests that tonsillar CXCR5⁺ cells may serve as a long-lived reservoir capable of seeding effector responses locally.

A particularly intriguing finding of our study was the pronounced enrichment of CXCR5 expression among EBV-specific memory CD8^+^ T cells in tonsil tissue. Of note, functional EBV-specific memory CD8^+^ T cells frequently expressed CXCR5 alongside the inhibitory receptors PD-1 and TIGIT, consistent with previous work (*41*). EBV-specific memory CD8^+^ T cells were also more prevalent in tonsil tissue compared with the vascular circulation, likely reflecting the presence of latent viral reservoirs in LTs. In addition, EBV-specific memory CD8^+^ T cells targeting latent antigens expressed CXCR5 more commonly than EBV-specific memory CD8^+^ T cells targeting lytic antigens, potentially as a consequence of differences in antigen expression. Lytic antigens are only expressed upon lytic reactivation, which is a rare event. After initial infection, EBV persists in memory B cells, where it limits its gene expression and only expresses a limited number of latent antigens. The dichotomy could also serve to optimize immune control of EBV. Specifically, active lytic infection requires a rapid cytotoxic response, whereas quiescent latent infection requires continuous immune surveillance (*42*). The enrichment of latent antigen–specific CXCR5^+^ memory CD8^+^ T cells in tonsil tissue likely accomplishes this latter role at a preferred site of viral persistence. The higher expression level of CD27 in tetramer^+^, tonsil-localized memory CD8^+^ T cells supports this, as CD27^+^ T cells have been associated with a protective role against EBV, and a recent study on human blood samples gave evidence for CD27 as a marker for EBV-specific T cells (*43*). Together with this, the pronounced enrichment of EBV latent antigen–specific cells within the same pool further emphasizes the role of tonsils as critical sites of EBV immunosurveillance. Since latent infection persists lifelong in B cells, the presence of stem-like CXCR5⁺ CD8⁺ T cells in close proximity to these reservoirs may be a key mechanism preventing viral reactivation and transformation. This niche may therefore represent a target for therapeutic reinforcement in settings of immune suppression, where EBV-driven lymphoproliferative disease emerges.

On the basis of these findings, it seems reasonable to propose that CXCR5^+^ memory CD8^+^ T cells are optimally configured and positioned to maintain localized immunity against latent infections with a predilection for LTs, epitomized here by EBV. In particular, tonsil-localized CXCR5^+^ memory CD8^+^ T cells exhibited features of stemness and tissue residency, with a noncytotoxic functional profile enriched for specificities derived from the immunodominant viral antigens EBNA3A and latent membrane protein 2 (LMP2). These adaptations at a major viral reservoir may inform vaccine strategies aimed at enhancing CD8⁺ T cell control of EBV, particularly in contexts of immune suppression where EBV-associated malignancies arise. More broadly, defining how tissue phenotypes shape T cell function will be critical for optimizing T cell–based therapies for site-specific efficacy.

## MATERIALS AND METHODS

### Sample collection

Peripheral blood and matched tonsil samples were obtained from individuals undergoing tonsillectomy for recurrent infections or obstructive sleep apnea (female, *n* = 22; male, *n* = 21) as part of the TONCIM Tissue Collection Programme at ÖNH Odenplan, Stockholm, Sweden. The study was approved by the Regional Ethics Committee in Stockholm (2021-03694). Peripheral blood and other matched tissue samples were obtained from deceased organ donors (female, *n* = 13; male, *n* = 24) as part of the IHOPE centered at Karolinska University Hospital, Stockholm, Sweden. Inclusion criteria followed national guidelines for organ donation and transplantation. Tissues not used for transplantation were donated after serological testing for CMV, hepatitis B virus core and surface antigens, HIV-1, and syphilis (*Treponema pallidum*). The study was approved by the Regional Ethics Committee in Stockholm (2019-05016). All participants or their legal representatives provided written informed consent in accordance with the principles of the Declaration of Helsinki. Donor characteristics are summarized in table S1.

### Sample processing

Tonsils were cleaned, cut into small pieces, ground, and filtered through a cell strainer (pore size = 100 μm). The cell suspension was then washed with phosphate-buffered saline (PBS) and filtered through another cell strainer (pore size = 70 μm). Mononuclear cells were cryopreserved in fetal bovine serum (FBS) containing 10% dimethyl sulfoxide (DMSO). Other tissues were rinsed with PBS and incubated in sterile petri dishes containing RPMI 1640 medium supplemented with 10% FBS, 1% l-glutamine, 1% penicillin/streptomycin (complete medium; Thermo Fisher Scientific), and deoxyribonuclease (DNase) I (10 U/ml; Sigma-Aldrich). After filtering through a cell strainer (pore size = 70 μm), cell suspensions were washed with PBS, dispensed at 1 × 10^6^ to 2 × 10^6^ cells per well in 96-well U-bottom plates (Corning), and stained after resting for 2 hours at 37°C. Some tissues were processed using additional steps after incubation. Gut components were cut into small pieces and digested for 1 hour at 37°C on an orbital shaker (120 rpm) in Falcon tubes containing complete medium supplemented with purified collagenase (80 μg/ml; Worthington Biochemical). Tissue pieces were then filtered through a cell strainer and washed as above. Skin samples were trimmed with scissors to remove subcutaneous fat and biopsied at a diameter of 5 mm. Biopsies were then digested for 1.5 hour at 37°C in complete medium supplemented with purified collagenase (240 μg/ml; Worthington Biochemical), filtered through a cell strainer, and washed as above. Peripheral blood mononuclear cells (PBMCs) were isolated via standard density gradient centrifugation using Ficoll-Paque Plus (Sigma-Aldrich) and cryopreserved in FBS containing 10% DMSO.

### Functional assays

Lyophilized pools of optimal overlapping peptides were reconstituted at a stock concentration of 10 mg/ml in DMSO, diluted to 100 μg/ml, and stored at −20°C. Overlapping peptide pools spanning the CMV proteins IE-1 and pp65, the EBV latent proteins EBNA3A and LMP2, and the EBV lytic proteins BRLF1, BZLF1, and gp350 were purchased from JPT Peptide Technologies. The relevant peptides were combined to generate single pools for CMV antigens, EBV latent antigens, and EBV lytic antigens. Cryopreserved PBMCs and tonsil cells were thawed quickly, resuspended in complete medium supplemented with DNase I (10 U/ml; Sigma-Aldrich), and rested at 1 to 2 × 10^6^ cells per well in 96-well U-bottom plates (Corning) for 2 hours at 37°C. Rested cells were washed with PBS and labeled with LIVE/DEAD Fixable Aqua (Thermo Fisher Scientific) for 10 min at room temperature. The medium was then supplemented with anti-CXCR5–APC, followed 15 min later by the relevant peptide pool (0.5 μg/ml per peptide). Negative control wells contained an equivalent volume of DMSO, and positive control wells contained staphylococcal enterotoxin B (0.5 μg/ml; Sigma-Aldrich). Brefeldin A (1 μg/ml; Sigma-Aldrich) and monensin (0.7 μg/ml; BD Biosciences) were added after 1 hour, followed by anti-CD107a–PE-Cy5. Cells were then incubated at for 6 hours at 37°C. After incubation, cells were washed with PBS containing 2% FBS and 2 mM EDTA [fluorescence-activated cell sorter (FACS) buffer] and stained with anti-CCR7–APC-Cy7 for 10 min at 37°C, followed by anti-CD3–BUV805, anti-CD4–PE-Cy5.5, anti-CD8–BUV396, anti-CD14–BV480, anti-CD19–BV480, anti-CD38–BUV496, anti-CD40L–BV421, anti-CD45RA–BV570, anti-CD49a–BUV615, anti-CD69–BUV563, anti-CD95–PE-CF594, anti-CD103–BV605, anti-PD-1–BUV661, anti-TIGIT–BUV737, and anti-41BB–PE-Cy7 for 30 min at room temperature. Cells were then washed with FACS buffer, fixed/permeabilized using a FoxP3 transcription factor staining buffer set (eBioscience), and stained with anti-GzmB–BB790, anti–IFN-γ–PE, anti–IL-2–AF700, anti–IL-17a–BV711, anti–TNF-α–BV650, and anti–TCF-1–BB515 for 60 min at room temperature. Stained cells were washed with FACS buffer, fixed in PBS containing 1% paraformaldehyde (PFA; Biotium), and acquired using a FACSymphony A5 (BD Biosciences). Flow cytometry reagents are listed in table S2.

### Ex vivo proliferation assays

Tonsil and blood samples were thawed and labeled with CellTrace Blue dye. Cells were cultured for 7 days in RPMI 1640 supplemented with 10% FBS, 1% penicillin-streptomycin, 1% l-glutamine, recombinant human IL-2 (150 U/ml), and IL-7 (50 U/ml). At day 0 and day 7, cells were stained with anti-CXCR5–PE and anti-CCR7–APC-Cy7 for 10 min at 37°C, followed by anti-CD3–BUV805, anti-CD4–PE-Fire710, anti-CD8–BV711, anti-CD14–BV510, anti-CD19–BV510, anti-CD38–BUV496, anti-CD45RA–BV570, anti-CD49a–BUV615, anti-CD69–BUV563, anti-CD95–PE-Dazzle594, anti-CD103–BV605, anti-CD127–PE-Cy5, anti-HLA-DR–BB700, and anti-PD-1–PE-Cy7 for 30 min at room temperature. Viable cells were identified by exclusion using a LIVE/DEAD Fixable Aqua Dead Cell Stain Kit (Thermo Fisher Scientific). Cells were then washed with FACS buffer, fixed, and permeabilized using the Intracellular Staining Kit (BioLegend) for 1 hour at room temperature. After washing, intracellular staining was performed with anti-TCF1–AF488, anti-Ki67–RB744, anti–GzmB–BB790, and anti-Granzyme K–RB780 for 30 min. Samples were acquired on a Cytek Aurora (Cytek). All flow cytometry reagents are listed in table S2.

### Tetramer staining

HLA class I tetramers were generated as described previously (*44*). The following specificities were used in this study: CMV NLVPMVATV/A02:01 (pp65), KLGGALQAK/A03:01 (IE-1), QYDPVAALF/A24:02 (pp65), RPHERNGFTVL/B07:02 (pp65), QIKVRVDMV/B08:01 (IE-1); EBV CLGGLLTMV/A02:01 (LMP2, latent), GLCTLVAML/A02:01 (BMLF1, lytic), RLRAEAQVK/A03:01 (EBNA3A, latent), RVRAYTYSK/A03:01 (BRLF1, lytic), TYGPVFMCL/A24:02 (LMP2, latent), RPPIFIRRL/B07:02 (EBNA3A, latent), RAKFKQLL/B08:01 (BZLF1, lytic), and Influenza GILGFVFTL/A02:01 (M1), ILRGSVAHK/A03:01 (NP), LPFDKTTVM/B07:02 (NP), and ELRSRYWAI/B08:01 (NP). Cryopreserved PBMCs and tonsil cells were thawed quickly, resuspended in complete medium supplemented with DNase I (10 U/ml; Sigma-Aldrich), dispensed at 1 × 10^6^ to 2 × 10^6^ cells per well in 96-well U-bottom plates (Corning), and labeled with a mix of BV421-conjugated and PE-conjugated tetramers in the presence of dasatinib (50 nM; STEMCELL Technologies) for 15 min at room temperature. Cells were then washed with FACS buffer and stained with anti-CCR5–APC, anti-CCR6–R718, anti-CCR7–APC-Cy7, anti-CXCR3–BB700, anti-CXCR5–BB515, and anti-CXCR6–PE-Dazzle 594 for 10 min at 37°C, followed by anti-CD3–BUV805, anti-CD4–PE-Cy5.5, anti-CD8–BUV395, anti-CD14–BV510, anti-CD19–BV510, anti-CD27–BV786, anti-CD38–BUV496, anti-CD39–BV711, anti-CD45RA–BV570, anti-CD49a–BUV615, anti-CD69–BUV563, anti-CD95–BB630, anti-CD101–BUV661, anti-CD103–BV605, anti-CD127–PE-Cy5, anti–HLA-DR–BV750, anti–PD-1–PE-Cy7, anti-TIGIT–BUV737, and anti–TIM-3–BV650 for 30 min at room temperature. Viable cells were identified by exclusion using a LIVE/DEAD Fixable Aqua Dead Cell Stain Kit (Thermo Fisher Scientific). Stained cells were washed with FACS buffer, fixed in PBS containing 1% PFA, and acquired using a FACSymphony A5 (BD Biosciences). Flow cytometry reagents are listed in table S2.

### Single-cell RNA-seq

Cryopreserved matched PBMCs and tonsil cells from 4 donors were thawed quickly, resuspended in complete medium supplemented with DNase I (10 U/ml; Sigma-Aldrich), and rested en masse for 3 hours at 37°C. Unwanted cells were then removed via magnetic separation using an EasySep Human T Cell Isolation Kit (STEMCELL Technologies). Isolated cells were stained with fluorophore-conjugated versions of the oligonucleotide-conjugated antibodies (TotalSeq; BioLegend) listed in table S3. Target cell populations were gated serially as lymphocytes/live/CD8^+^/non-CCR7^+^CD45RA^+^ events, and CXCR5^+^ and CXCR5^−^ cells (*n* = 15,000 per population) were sorted via flow cytometry in UltraPurity mode using an MA900 Cell Sorter (Sony). Cells from each sample were then pooled and processed using Chromium Next GEM Single Cell V(D)J Reagent Kits version 1.1 (Rev G; 10x Genomics). Libraries were sequenced using a NovaSeq6000 (Illumina). Output data were processed using Cell Ranger version 7.0.0 (10x Genomics).

### Flow cytometry data analysis

Flow cytometry data were compensated using single-stained CompBeads (BD Biosciences) and analyzed using FlowJo software version 10.8 (FlowJo LLC). The gating strategy is shown in fig. S1. Cell frequencies correspond to the parent gates unless specified otherwise. Statistical differences between groups were assessed using the Mann-Whitney *U* test or the Wilcoxon signed-rank test. Activated and antigen-specific cell populations were excluded from the analysis below a threshold of 10 activation marker^+^ or tetramer^+^ cells per population, respectively. PCAs were performed using the prcomp function with scaling in R. Heatmaps were generated using median percentage values for each cell population.

### Single-cell profiling analysis

CellRanger outputs were analyzed using Seurat version 4.3.0. Samples were demultiplexed using the HTODemux function in R. Cells were filtered to exclude those expressing <500 genes and those with a mitochondrial content of >10%. UMAP visualization was conducted across 15 dimensions, and clusters were identified using a resolution of 0.2. Cluster markers were identified using the FindAllMarkers function with a logFC threshold of 0.25 and a minimum expressed percentage of 0.25. Differential expression analysis was performed using the Wilcoxon rank-sum test with a minimum expressed percentage of 0.2. Gene set enrichment analysis was performed using the gsea package with 5000 permutations and gene sets downloaded from the Molecular Signatures Database (MSigDB) using msigdbr version 7.5.1. Module scores were calculated using the AddModuleScore function in Seurat version 4.3.0. Cells with at least one reconstructed chain were included for clonotype analysis, and clonality was assigned on the basis of amino acid sequence identity across the third complementarity-determining region (CDR3).
