# Supplementary material for: CXCR5 identifies stem-like resident memory CD8⁺ T cells enriched for latent EBV specificity in tonsils
Source: Sci Adv. 2026 Jan 7;12(2):eady8316. doi: 10.1126/sciadv.ady8316 (PMC12778062; doi:10.1126/sciadv.ady8316)
Supplement: Supplementary file 1 — Figs. S1 to S8 Tables S1 to S3 [file sciadv.ady8316_sm.pdf]

Science Advances
AAAS

# Supplementary Materials for

## CXCR5 identifies stem-like resident memory CD8+ T cells enriched for latent EBV specificity in tonsils

Olga Rivera Ballesteros *et al.*

Corresponding author: Marcus Buggert, marcus.buggert@ki.se

*Sci. Adv.* **12**, eady8316 (2026)
DOI: 10.1126/sciadv.ady8316

**This PDF file includes:**

Figs. S1 to S8
Tables S1 to S3

## SUPPLEMENTARY MATERIAL

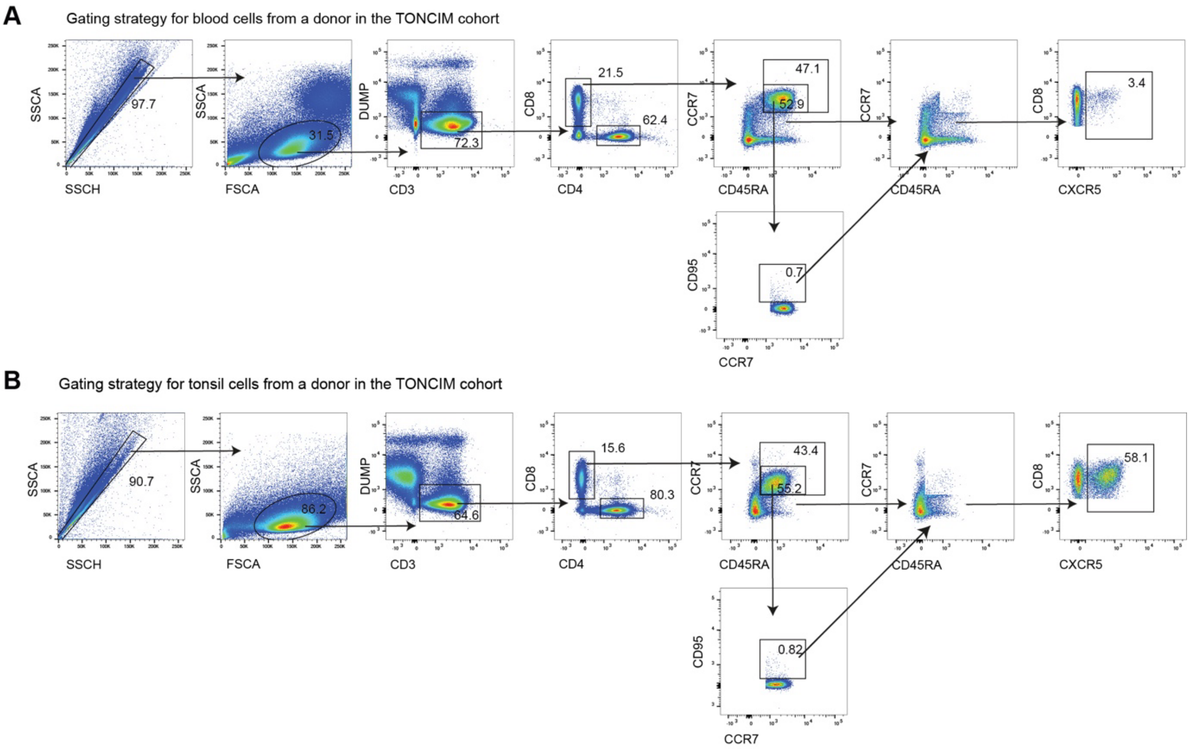


**Supplementary Fig. S1. Gating strategy for the identification of CXCR5$^+$ memory CD8$^+$ cells in blood and tonsil tissue.** Representative flow cytometry plots showing the identification of CXCR5$^+$ memory CD8$^+$ T cells in blood (**A**) and tonsil tissue (**B**). Memory populations were captured in a composite gate encompassing $T_{SCM}$ cells (CCR7$^+$CD45RA$^+$CD95$^+$), $T_{CM}$ cells (CCR7$^+$CD45RA$^-$), $T_{EM}$ cells (CCR7$^-$CD45RA$^-$), and $T_{EMRA}$ cells (CCR7$^-$CD45RA$^+$).

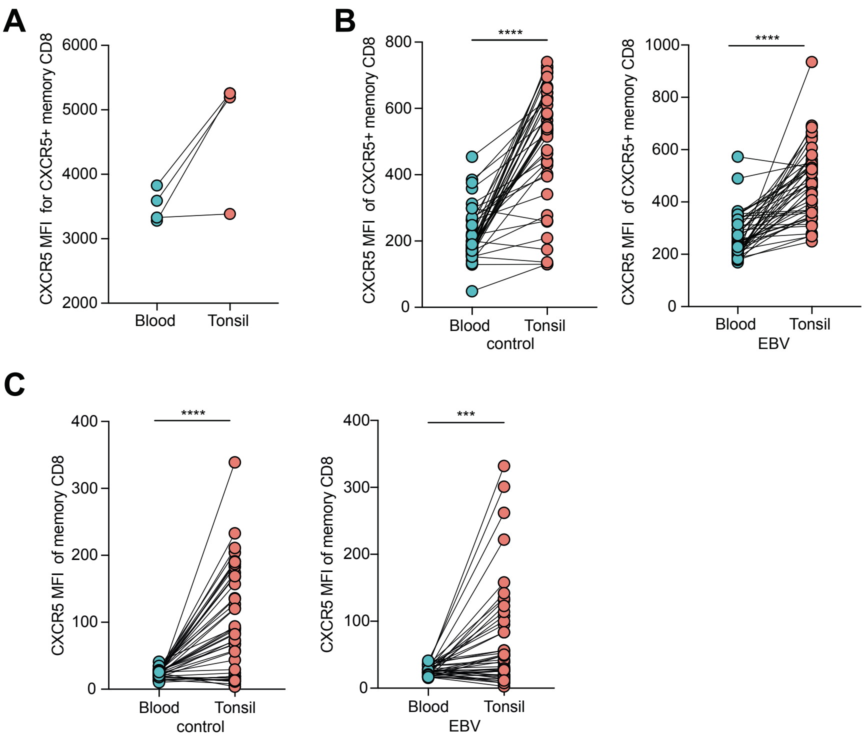


**Supplementary Fig. S2. CXCR5 expression levels.** Expression levels of CXCR5 visualized as Median Fluorescence Intensity (MFI). (**A**) CXCR5 MFI of CXCR5$^{+}$ memory CD8$^{+}$ T cells in blood and tonsil for the donors shown in Fig 2 and Fig 3. (**B**) CXCR5 MFI of CXCR5$^{+}$ memory CD8$^{+}$ T cells in blood and tonsil for control and EBV peptide stimulated cells. (**C**) CXCR5 MFI of memory CD8$^{+}$ T cells in blood and tonsil for control and EBV peptide stimulated cells. Paired t-test and Wilcoxon Rank Sum Test; ***p<0.001, ****p<0.0001

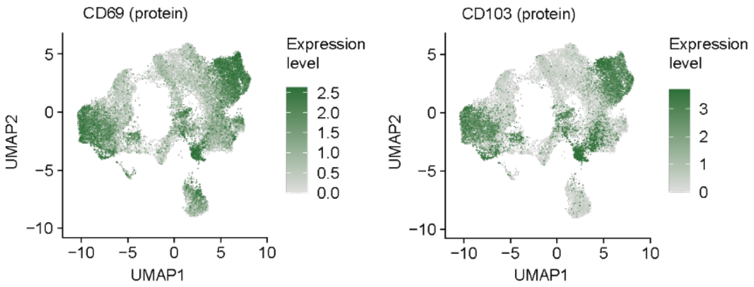


**Supplementary Fig. S3. Markers of tissue residency expressed at the protein level among CXCR5$^+$ and CXCR5$^-$ memory CD8$^+$ T cells in tonsil tissue.** UMAP visualization of CD69 and CD103 protein expression in tonsil tissue.

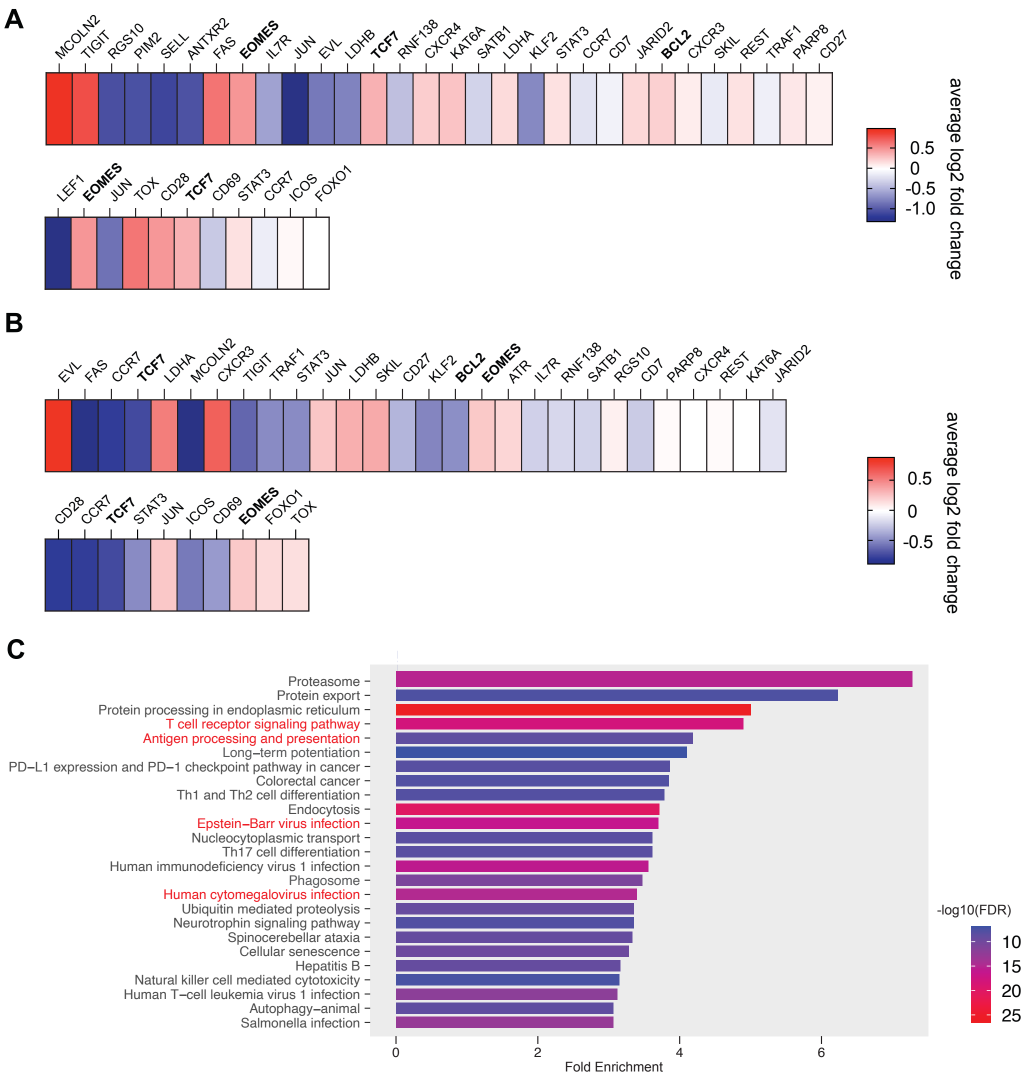


**Supplementary Fig. S4. CXCR5$^{+}$ T cell transcriptomics.** Comparison of Differentially Expressed (DE) Genes from (**A**) CXCR5$^{+}$ versus CXCR5$^{-}$ memory CD8$^{+}$ T cells from tonsil and (**B**) Blood versus Tonsil isolated memory CD8$^{+}$ T cells with literature stem-like T cell signatures. Upper panel shows average log2FC value for genes overlapping with stem-like T cell signature from Gueguen et al (*41*) and lower panel with stem-like markers from Steiner et al (*42*). (**C**) Top 25 enriched pathways (KEGG) for upregulated genes comparing CXCR5$^{+}$ versus CXCR5$^{-}$ memory CD8$^{+}$ T cells from tonsil.

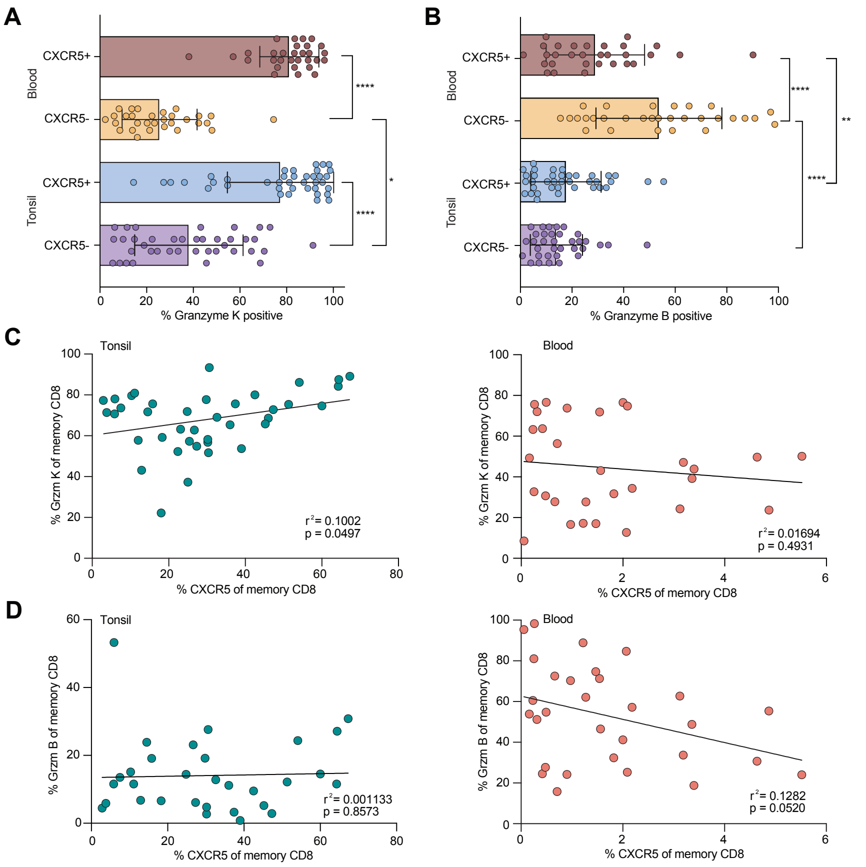


**Supplementary Fig. S5. Granzyme K expression in memory CD8⁺ T cells from blood and tonsil.** Expression of granzyme K (**A**) and granzyme B (**B**) in blood and tonsils for CXCR5⁺ and CXCR5⁻ memory CD8⁺ T cells. Correlation analysis of granzyme K (**C**) or granzyme B (**D**) and CXCR5⁺ expression within memory CD8⁺ T cell population in tonsil and blood. (**A,B**) n=37-39 patient samples; MWU test * p<0.05, ** p<0.01, **** p<0.0001. (**C,D**) n=30-39 patient samples; simple linear regression

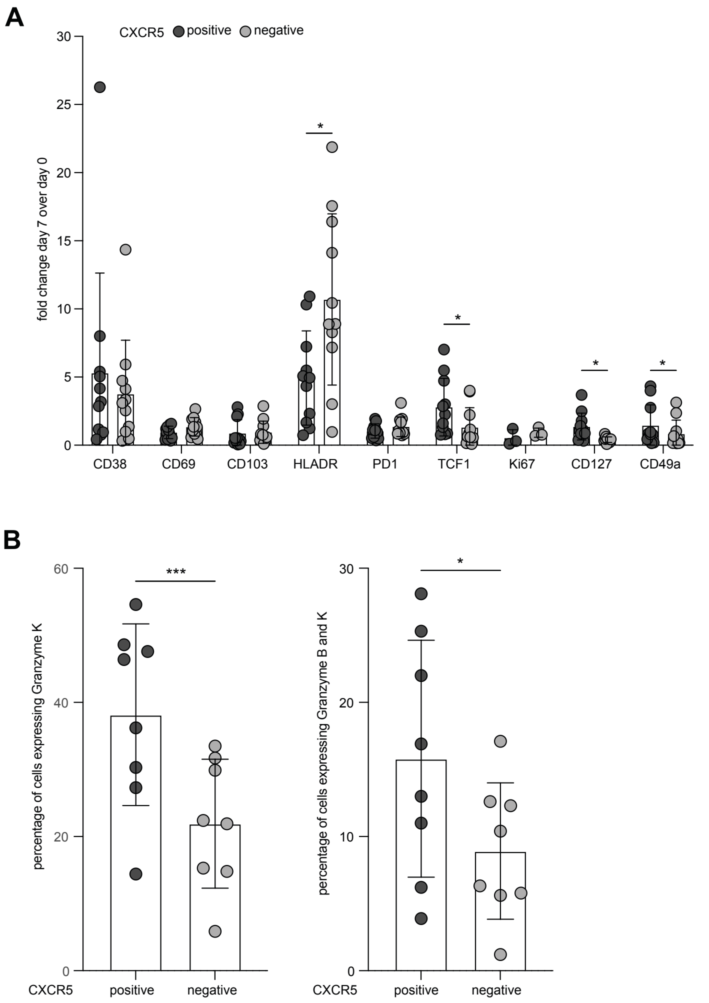


**Supplementary Fig. S6. Tracking CXCR5$^{+}$ and CXCR5$^{-}$ cells over time.** Tonsil cells were labeled with Cell Trace Blue, cultured for 7 days and phenotypic changes of CXCR5$^{+}$ and CXCR5$^{-}$ memory CD8$^{+}$ populations were followed. (**A**) Phenotypic changes of CXCR5 positive and negative populations at d7 compared to d0 as fold change. (**B**) Expression of Granzyme K and B in CXCR5 positive and negative populations. Paired t-test *$p<0.05$, ***$p<0.001$; N=2 independent experiments with n=3-11 donors.

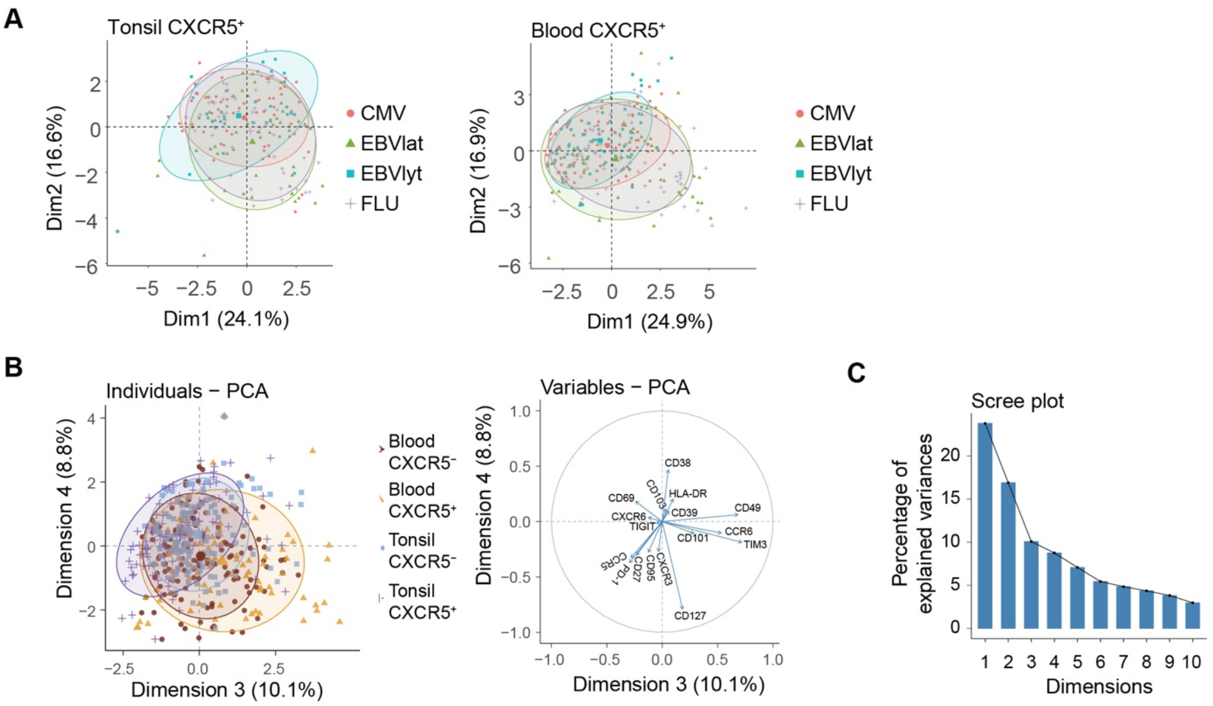


**Supplementary Fig. S7. Principal component analysis of virus-specific memory CD8⁺ T cell phenotypes in blood and tonsil tissue.** (**A**) PCA projections showing tetramer⁺ CXCR5⁺ memory CD8⁺ T cells segregated by the expression of phenotypic markers measured via flow cytometry. Populations from blood and tonsil samples are colored by specificity. (**B**) PCA projections showing tetramer⁺ memory CD8⁺ T cells segregated by the expression of phenotypic markers measured via flow cytometry across dimensions 3 and 4. Left: populations are colored by CXCR5 expression and tissue origin. Right: individual markers are shown by relative contribution. (**C**) Scree plot showing explained variance across the first ten dimensions from analyses of tetramer⁺ memory CD8⁺ T cells segregated by the expression of CXCR5.

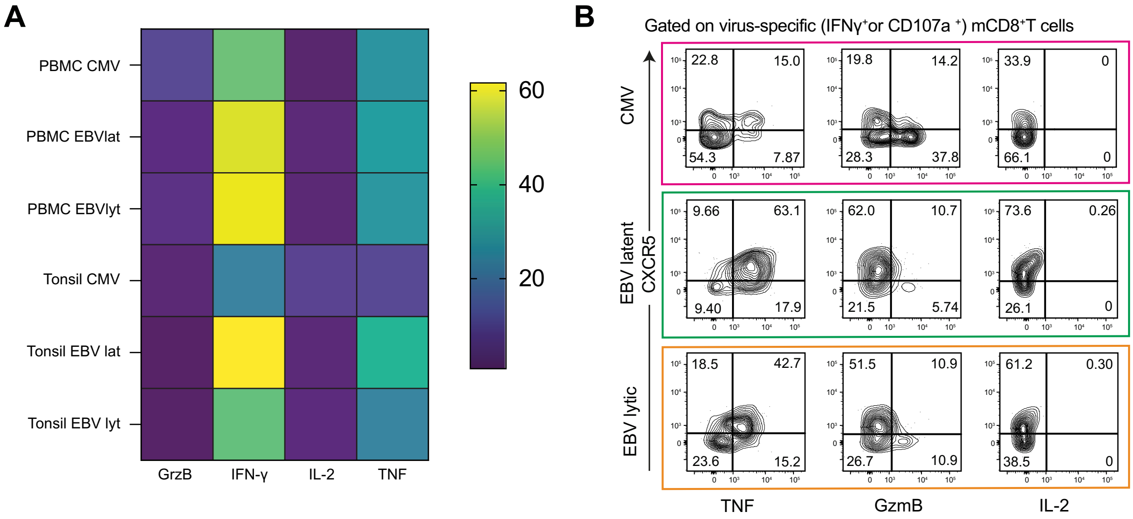


**Supplementary Fig. S8. Cytokine production among virus-specific memory CD8⁺ T cells in blood and tonsil tissue.** (**A**) Heatmap showing the median expression of granzyme B (GzmB), IFN- γ, IL-2 or TNF among functional (CD107a⁺ or IFN-γ⁺) virus-specific CXCR5⁺ memory CD8⁺ T cells in blood and tonsil tissue. (**B**) Gating strategy for TNF, GrzB and IL-2 shown for one sample per peptide stimulation condition (CMV, EBVlatent, EBVlytic).

**Table S1. Donor characteristics.**

| Donor | Age | Sex | Cohort | CMV | EBV | Reason for surgery |
|---|---|---|---|---|---|---|
| IHOPE2 | 67 | M | IHOPE | + | ND | NA |
| IHOPE4 | 83 | F | IHOPE | + | + | NA |
| IHOPE5 | 61 | M | IHOPE | + | + | NA |
| IHOPE6 | 69 | M | IHOPE | + | + | NA |
| IHOPE7 | 84 | M | IHOPE | + | + | NA |
| IHOPE9 | 66 | M | IHOPE | + | + | NA |
| IHOPE11 | 65 | M | IHOPE | + | + | NA |
| IHOPE12 | 68 | M | IHOPE | + | + | NA |
| IHOPE14 | 80 | M | IHOPE | + | + | NA |
| IHOPE18 | 69 | M | IHOPE | + | + | NA |
| IHOPE27 | 80 | M | IHOPE | + | + | NA |
| IHOPE28 | 62 | M | IHOPE | + | + | NA |
| IHOPE30 | 61 | M | IHOPE | + | ND | NA |
| IHOPE31 | 85 | F | IHOPE | + | ND | NA |
| IHOPE32 | 76 | F | IHOPE | + | + | NA |
| IHOPE34 | 68 | F | IHOPE | + | ND | NA |
| IHOPE35 | 61 | M | IHOPE | - | + | NA |
| IHOPE36 | 43 | F | IHOPE | - | + | NA |
| IHOPE38 | 32 | F | IHOPE | + | + | NA |
| IHOPE39 | 68 | M | IHOPE | + | ND | NA |
| IHOPE40 | 64 | M | IHOPE | + | + | NA |
| IHOPE41 | 42 | F | IHOPE | + | + | NA |
| IHOPE42 | 58 | F | IHOPE | + | + | NA |
| IHOPE43 | 68 | M | IHOPE | + | ND | NA |
| IHOPE44 | 54 | M | IHOPE | + | + | NA |
| IHOPE45 | 50 | M | IHOPE | + | ND | NA |
| IHOPE47 | 62 | M | IHOPE | + | + | NA |
| IHOPE48 | 65 | F | IHOPE | + | + | NA |
| IHOPE49 | 65 | M | IHOPE | - | + | NA |
| IHOPE50 | 71 | F | IHOPE | + | + | NA |
| IHOPE51 | 74 | F | IHOPE | + | ND | NA |
| IHOPE52 | 56 | F | IHOPE | - | + | NA |
| IHOPE53 | 53 | M | IHOPE | + | ND | NA |
| IHOPE54 | 64 | M | IHOPE | + | ND | NA |
| IHOPE55 | 77 | M | IHOPE | + | - | NA |
| IHOPE56 | 64 | M | IHOPE | + | + | NA |
| IHOPE58 | 41 | F | IHOPE | + | ND | NA |
| TONCIM002 | 3 | M | TONCIM | ND | ND | OSA |
| TONCIM003 | 5 | M | TONCIM | ND | ND | OSA |
| TONCIM004 | 20 | F | TONCIM | ND | ND | Recurrent tonsillitis |
| TONCIM005 | 17 | F | TONCIM | ND | ND | Recurrent tonsillitis |
| TONCIM006 | 4 | F | TONCIM | ND | ND | Hypertrophy |
| TONCIM007 | 5 | M | TONCIM | ND | ND | Hypertrophy |
| TONCIM008 | 6 | F | TONCIM | ND | ND | Hypertrophy |
| TONCIM009 | 13 | F | TONCIM | ND | ND | Recurrent tonsillitis |
| TONCIM010 | 15 | M | TONCIM | ND | ND | Recurrent tonsillitis |
| TONCIM011 | 21 | M | TONCIM | ND | ND | Recurrent tonsillitis |
| TONCIM017 | 30 | M | TONCIM | ND | ND | NA |
| TONCIM018 | 19 | F | TONCIM | ND | ND | Recurrent tonsillitis |
| TONCIM019 | 22 | F | TONCIM | ND | ND | Recurrent tonsillitis |
| TONCIM020 | 27 | F | TONCIM | ND | ND | Recurrent tonsillitis |
| TONCIM021 | 24 | F | TONCIM | ND | ND | Hypertrophy |
| TONCIM022 | 18 | F | TONCIM | ND | ND | Recurrent tonsillitis |
| TONCIM029 | 23 | F | TONCIM | ND | ND | Recurrent tonsillitis |
| TONCIM030 | 20 | F | TONCIM | ND | ND | Recurrent tonsillitis |

| | | | | | | |
|---|---|---|---|---|---|---|
| TONCIM034 | 15 | F | TONCIM | ND | ND | NA |
| TONCIM036 | 28 | F | TONCIM | ND | ND | NA |
| TONCIM042 | 12 | F | TONCIM | ND | ND | PF-APA |
| TONCIM043 | 17 | M | TONCIM | ND | ND | Recurrent tonsillitis |
| TONCIM044 | 29 | M | TONCIM | ND | ND | Recurrent tonsillitis |
| TONCIM045 | 38 | M | TONCIM | ND | ND | Recurrent tonsillitis |
| TONCIM046 | 15 | F | TONCIM | ND | ND | Recurrent tonsillitis |
| TONCIM047 | 18 | F | TONCIM | ND | ND | Recurrent tonsillitis |
| TONCIM051 | 22 | F | TONCIM | ND | ND | Peritonsillitis |
| TONCIM058 | 31 | F | TONCIM | ND | ND | Recurrent tonsillitis |
| TONCIM059 | 29 | F | TONCIM | ND | ND | OSA |
| TONCIM060 | 13 | F | TONCIM | ND | ND | Recurrent tonsillitis |
| TONCIM068 | 20 | F | TONCIM | ND | ND | Hypertrophy + Recurrent tonsillitis |
| TONCIM069 | 24 | M | TONCIM | ND | ND | Recurrent tonsillitis |
| TONCIM071 | 17 | F | TONCIM | ND | ND | Recurrent tonsillitis |
| TONCIM072 | 31 | M | TONCIM | ND | ND | Chronic tonsillitis |
| TONCIM074 | 44 | M | TONCIM | ND | ND | Recurrent sore throat |
| TONCIM075 | 11 | F | TONCIM | ND | ND | Chronic tonsillitis |
| TONCIM083 | 20 | F | TONCIM | ND | ND | Tonsillitis |
| TONCIM084 | 19 | F | TONCIM | ND | ND | Recurrent tonsillitis |
| TONCIM097 | 26 | M | TONCIM | ND | ND | Recurrent tonsilitis |
| TONCIM099 | 19 | M | TONCIM | ND | ND | Chronic tonsillitis |
| TONCIM103 | 18 | F | TONCIM | ND | ND | Periodic fever |
| TONCIM104 | 23 | F | TONCIM | ND | ND | Chronic tonsillitis |
| TONCIM107 | 29 | M | TONCIM | ND | ND | Recurrent tonsillitis |
| TONCIM109 | 21 | M | TONCIM | ND | ND | Chronic tonsillitis |
| TONCIM113 | 31 | M | TONCIM | ND | ND | OSAS |
| TONCIM114 | 15 | M | TONCIM | ND | ND | Chronic tonsillitis |
| TONCIM121 | 19 | F | TONCIM | ND | ND | Recurrent tonsillitis |
| TONCIM122 | 28 | M | TONCIM | ND | ND | Recurrent tonsillitis |
| TONCIM123 | 20 | F | TONCIM | ND | ND | Recurrent tonsillitis |
| TONCIM129 | 17 | M | TONCIM | ND | ND | Chronic tonsillitis |
| TONCIM140 | 18 | M | TONCIM | ND | ND | Recurrent tonsillitis |
| TONCIM144 | 25 | M | TONCIM | ND | ND | Chronic tonsillitis |
| TONCIM146 | 22 | F | TONCIM | ND | ND | Recurrent tonsillitis |
| TONCIM149 | 32 | F | TONCIM | ND | ND | Chronic tonsillitis |
| TONCIM151 | 34 | F | TONCIM | ND | ND | Recurrent tonsillitis |
| TONCIM154 | 13 | F | TONCIM | ND | ND | Chronic tonsillitis |
| TONCIM155 | 36 | M | TONCIM | ND | ND | Recurrent tonsillitis |
| TONCIM157 | 15 | M | TONCIM | ND | ND | Chronic tonsillitis |
| TONCIM158 | 17 | M | TONCIM | ND | ND | Chronic tonsillitis |
| TONCIM160 | 17 | F | TONCIM | ND | ND | Chronic tonsillitis |
| TONCIM162 | 23 | F | TONCIM | ND | ND | Recurrent tonsillitis |
| TONCIM163 | 21 | F | TONCIM | ND | ND | Chronic tonsillitis |
| TONCIM164 | 21 | F | TONCIM | ND | ND | Recurrent tonsillitis |
| TONCIM165 | 22 | F | TONCIM | ND | ND | Chronic tonsillitis |
| TONCIM166 | 29 | F | TONCIM | ND | ND | Recurrent tonsillitis |
| TONCIM167 | 31 | F | TONCIM | ND | ND | Recurrent tonsillitis |
| TONCIM169 | 4 | M | TONCIM | ND | ND | OSAS |
| TONCIM170 | 17 | M | TONCIM | ND | ND | Chronic tonsillitis |
| TONCIM171 | 30 | M | TONCIM | ND | ND | Chronic tonsillitis |
| TONCIM172 | 24 | F | TONCIM | ND | ND | Chronic tonsillitis |
| TONCIM173 | 24 | F | TONCIM | ND | ND | Chronic tonsillitis |
| TONCIM191 | 17 | M | TONCIM | ND | ND | Chronic tonsillitis |
| TONCIM193 | 19 | F | TONCIM | ND | ND | Chronic tonsillitis |

NA, not applicable; ND, not done; OSAS, obstructive sleep apnea syndrome.

**Table S2. Flow cytometry reagents and analysis software.**

| Reagent | Catalog ID | Source | RRID |
|---|---|---|---|
| Anti-4-1BB–PE-Cy7 (4B4-1) | 309818 | BioLegend | AB_2562082 |
| Anti-CCR5–APC (2D7) | 2D7 | BioLegend | AB_2075402 |
| Anti-CCR6–R718 (11A9) | 11A9 | BioLegend | AB_10665163 |
| Anti-CD3–BUV805 (SK7) | 612892 | BD Biosciences | AB_2870076 |
| Anti-CD4–PECy5.5 (RPA-T4) | 300518 | BioLegend | AB_2870078 |
| Anti-CD4-PEFire710 (SK3) | 344665 | BioLegend | AB_2876651 |
| Anti-CD8–BUV396 (RPA-T8) | 563795 | BD Biosciences | AB_2737704 |
| Anti-CD8-BV711 (RPA-T8) | 301043 | BioLegend | AB_11218793 |
| Anti-CD14–BV510 (M5E2) | 301828 | BioLegend | AB_11219593 |
| Anti-CD19–BV510 (SJ25C1) | 659455 | BD Biosciences | AB_2562081 |
| Anti-CD27–BV786 (O323) | 302835 | BioLegend | AB_2562591 |
| Anti-CD38–BUV496 (HIT2) | 563969 | BD Biosciences | AB_2870082 |
| Anti-CD39–BV711 (eBioA1) | 344818 | BioLegend | AB_2562087 |
| Anti-CD40L–BV421 (24-31) | 563941 | BD Biosciences | AB_2870075 |
| Anti-CD45RA–BV570 (HI100) | 304134 | BioLegend | AB_2562622 |
| Anti-CD49a–BUV615 (TS2/7) | 742620 | BD Biosciences | AB_2870080 |
| Anti-CD69–BUV563 (FN50) | 741978 | BD Biosciences | AB_2870079 |
| Anti-CD95–BB630 (DX2) | 740805 | BD Biosciences | AB_2870081 |
| Anti-CD95–PE-CF594 (DX2) | 562396 | BD Biosciences | AB_2737718 |
| Anti-CD101–BUV661 (BB27) | 566292 | BD Biosciences | AB_2870077 |
| Anti-CD103–BV605 (Ber-ACT8) | 350207 | BioLegend | AB_2563660 |
| Anti-CD107a–PE-Cy5 (H4A3) | 555802 | BD Biosciences | AB_396136 |
| Anti-CD127–PE-Cy5 (A019D5) | 337108 | BioLegend | AB_2562088 |
| Anti-CXCR3–BB700 (1C6) | 566532 | BD Biosciences | AB_11163913 |
| Anti-CXCR5–APC (J252D4) | 356904 | BioLegend | AB_2561812 |
| Anti-CXCR5–BB515 (J252D4) | 564624 | BD Biosciences | AB_2738871 |
| Anti-CXCR6–PE/Dazzle594 (5A9) | 5A9 | BioLegend | AB_2814400 |
| Anti-GzmB–BB790 (GB12) | 372211 | BioLegend | AB_2562086 |
| Anti-GzmK-RB780 (G3H69) | 569226 | BD Biosciences | AB_3099638 |
| Anti-HLA-DR–BV750 (L243) | 307654 | BioLegend | AB_2563544 |
| Anti-HLA-DR-BB700 (G46-6) | 566481 | BD Biosciences | AB_2744477 |
| Anti-IFN-γ–PE (4S.B3) | 500308 | BioLegend | AB_2562083 |
| Anti-IL-2–AF700 (MQ1-17H12) | 500311 | BioLegend | AB_2562084 |
| Anti-IL-17A–BV711 (BL168) | 512328 | BioLegend | AB_2562085 |
| Anti-Ki67-RB744 (B56) | 570502 | BD Biosciences | AB_3685795 |
| Anti-PD-1–PE-Cy7 (EH12.1) | 329908 | BioLegend | AB_1953298 |
| Anti-TCF1–BB515 (C63D9) | 563020 | BD Biosciences | AB_2870087 |
| Anti-TCF1-AF488 (C63D9) | 6444S | Cell Signaling | AB_2797627 |
| Anti-TIGIT–BUV737 (1G9) | 744903 | BD Biosciences | AB_2870083 |
| Anti-TIM-3–BV650 (F38-2E2) | 345013 | BioLegend | AB_2562590 |
| Anti-TNF-α–BV650 (MAb11) | 502931 | BioLegend | AB_2562089 |
| BD GolgiStop | 554724 | BD Biosciences | AB_2869012 |
| Brefeldin A | 420601 | BioLegend | NA |
| Brilliant Stain Buffer Plus | 566385 | BD Biosciences | AB_2869761 |
| Dasatinib | 73082 | STEMCELL | NA |
| DNase I | 4716728001 | Sigma-Aldrich | NA |
| EasySep Human T Cell Isolation Kit | 17951 | STEMCELL | NA |
| FoxP3 Transcription Factor Staining Buffer Set | 00-5523-00 | Thermo Fisher Scientific | NA |
| LIVE/DEAD Fixable Aqua Dead Cell Stain Kit | L34957 | Thermo Fisher Scientific | NA |
| Paraformaldehyde | 22023 | Biotium | NA |
| PepMix Collection EBV | PM-C-EBV-2 | JPT | NA |
| PepMix Collection Human CMV | PM-C-HCMV-2 | JPT | NA |
| Purified collagenase (CLSPA) | LS005273 | Worthington | NA |

| | | | |
|---|---|---|---|
| Staphylococcal enterotoxin B | S4881 | Sigma-Aldrich | NA |
| FlowJo | NA | FlowJo LLC | SCR_008520 |
| RStudio | NA | Posit | SCR_001905 |

NA, not applicable.

**Table S3. CITE-seq reagents.**

| Reagent | Catalog ID | Source |
|---|---|---|
| TotalSeq-C0251 anti-human Hashtag 1 | 394661 | BioLegend |
| TotalSeq-C0252 anti-human Hashtag 2 | 394663 | BioLegend |
| TotalSeq-C0253 anti-human Hashtag 3 | 394665 | BioLegend |
| TotalSeq-C0254 anti-human Hashtag 4 | 394667 | BioLegend |
| TotalSeq-C0154 anti-human CD27 | 302853 | BioLegend |
| TotalSeq-C0386 anti-human CD28 | 302963 | BioLegend |
| TotalSeq-C0389 anti-human CD38 | 303543 | BioLegend |
| TotalSeq-C0087 anti-human CD45RO | 304259 | BioLegend |
| TotalSeq-C0147 anti-human CD62L | 304851 | BioLegend |
| TotalSeq-C0146 anti-human CD69 | 310951 | BioLegend |
| TotalSeq-C0394 anti-human CD71 | 334125 | BioLegend |
| TotalSeq-C0156 anti-human CD95 | 305651 | BioLegend |
| TotalSeq-C0145 anti-human CD103 | 350233 | BioLegend |
| TotalSeq-C0246 anti-human CD122 | 339021 | BioLegend |
| TotalSeq-C0390 anti-human CD127 | 351356 | BioLegend |
| TotalSeq-C0140 anti-human CD183 (CXCR3) | 353747 | BioLegend |
| TotalSeq-C0804 anti-human CD186 (CXCR6) | 356023 | BioLegend |
| TotalSeq-C0141 anti-human CD195 (CCR5) | 359137 | BioLegend |
| TotalSeq-C0088 anti-human CD279 (PD-1) | 329963 | BioLegend |
| TotalSeq-C0179 anti-human CX3CR1 | 355705 | BioLegend |
| TotalSeq-C0159 anti-human HLA-DR | 307663 | BioLegend |
